# Microbial seed coating: An attractive tool for sustainable agriculture

**DOI:** 10.1016/j.btre.2023.e00781

**Published:** 2023-01-05

**Authors:** Arezoo Paravar, Ramin Piri, Hamidreza Balouchi, Ying Ma

**Affiliations:** a Department of Crop Production and Plant Breeding, College of Agriculture, Shahed University, Tehran, Iran; b Department of Agronomy and Plant Breeding, College of Agriculture, University of Tehran, Tehran, Iran; c Department of Agronomy and Plant Breeding, Faculty of Agriculture, Yasouj University, Yasouj, Iran; d College of Resources and Environment, Southwest University, Chongqing, China

**Keywords:** Beneficial microorganisms, Agricultural sustainability, Delivery systems, Seed coating, Seed quality

## Abstract

Seed coating is considered one of the best methods to promote sustainable agriculture where the physical and physiological properties of seeds can be improved to facilitate planting, increase growth indices and alleviate abiotic and biotic stresses. Several methods of seed coating are used to attain good application uniformity and adherence in the seed coating process. Seed coating has been tested in seeds of various plant species with different dimensions, forms, textures, and germination types. Plant beneficial microorganisms (PBM), such as rhizobia, bacteria, and fungi inoculated via seed inoculation can increase seed germination, plant performance and tolerance across biotic (e.g., pathogens and pests) and abiotic stress (e.g., salt, drought, and heavy metals) while reducing the use of agrochemical inputs. In this review, the microbial seed coating process and their ability to increase seed performance and protect plants from biotic and abiotic stresses are well discussed and highlighted in sustainable agricultural systems.

## Highlights

• PBM include plant growth-promoting bacteria, mycorrhizal fungi and rhizobia.
• Seed coating with PBM is an effective on fertility of soils and health of plants.
• Innovative seed coating technology is provided for the delivery of many materials.
• Different methods are used in seed coating to attain good uniformity and adherence.

**Abbreviations**
PBM: Plant beneficial microorganisms
PGPB: Plant growth-promoting bacteria
AMF: Arbuscular mycorrhizal fungi

## 1 Introduction

Conventional agriculture is large-scale agriculture that widely uses artificial fertilizers, herbicides, and pesticides [1]. As an alternative, sustainable agriculture is fixed on management planes addressing the main societal worries about food quality or environmental protection. It involves two approaches: 1) agriculture should keep itself over a long period by conserving its productive resources, such as soil fertility maintenance, protection of groundwater, development of renewable energies, and detection solutions for acclimating farming systems to variations of climate; 2) agriculture systems should aid the sustainability of large domains and social societies [2]. Nowadays, without reducing the yield and quality of crops, the agricultural systems should apply minimum inputs and resources to attain economic advantagability, environmental security and social justice [3]. During the past ten years, the world population has considerably enhanced and is envisaged to attain around 9.5 billion by 2050 [4]. Therefore, given the growing global population, achieving global food security is possible through the design of advanced agricultural systems that can maximize our productivity and production with the minimum input required [5]. The additions contain phosphorus and nitrogen as fertilizer and pesticides as biocontrol agents for invasive weeds, pathogens, and insects. To increase or maintain crop yield, the farmers can benefit from new sustainable products, such as plant beneficial microorganisms (PBM) [6].

The soils are considered the densest and most diverse microbial habitats of plants [7]. Plant roots interact closely with soil micro-organisms. Complex interactions between roots and their related microbiomes are key factors in plant health [8]. The soil-borne pathogens limit and reduce plant growth, while the association of plants with PBM can promote plant growth. These PBM can facilitate plant nutrient uptake or increase stress tolerance [9]. In addition, they can protect plants against pathogens through antagonism, competition, and stimulation of the plant's immune system [9]. PBM include rhizobia associated with legumes and mycorrhizal fungi, as well as other free-living plant growth-promoting bacteria (PGPB) and fungi (PGPF) that and fungi (PGPF) that benefit a broad variety of plant species [10]. PBM can increase plant growth, facilitate water and nutrient uptake and distribution through different mechanisms [11]. For example, mycorrhizal symbiosis in soils may help to absorb and transfer water and nutrients through hyphae from the outer mycelium [12]. PGPB can help establish a root system and enhance plant growth by synthesizing bioactive substances such as phytohormones (e.g., auxins, gibberellins, and cytokinins), siderophore, and 1-aminocyclopropane-1-carboxylase (ACC) deaminase [12]. Moreover, nitrogen fixation through PBM occurs in free-living or non-coexistence (e.g., *Azotobacter*), coexistence (e.g., *Rhizobium*), and cooperation (e.g., *Azospirillum*) forms [13,14].

In the 1930s, the first seed coating artificial on cereal seeds was inspired by the pharmaceutical industry, and thereafter the using large-scale commercial of this tool started in the 1960s [15]. Nowadays, this tool was availed worldwide in horticultural and crop industries [14]. In the artificial seed coating, different materials (e.g., biopolymers, colorants, biocontrol agents, and microbes) are used in coating the surface of seeds [[14], [16]] to correct the physical features of seed crops and vegetable species, turfgrass, pasture, and flowers via deformation of seed weight and size [17]. The function of seed coating according to its mode of action or properties includes protecting plants, reducing environmental stress, or improving plant growth [18]. Indeed, seed coating is used as a biological tool that improves follow ability for agricultural sustainability [[14], [16]]. Considering these advantages, nowadays the application of this tool has been proposed for seed inoculation in different plants since it can use partial rates of inoculant in a precise use [19].

Hereby, the purpose of this review is to investigate the potential of plant beneficial microorganisms that are currently used as tools in seed coating processes and as an agent for improving physiological properties and seed germination and have more important for a sustainable agriculture society.

## 2 Plant beneficial microorganisms

### 2.1 Types of PBM

PBM are known as microorganisms that can increase plant establishment, growth and development, and defend plants across disease and abiotic stresses. PBM mainly include PGPB, arbuscular mycorrhizal fungi (AMF), and rhizobia.

#### 2.1.1 Plant growth-promoting bacteria

The most PBM present in soils are PGPB such as *Azospirillum, Azotobacter, Pseudomonas*, and *Bacillus*, which are bacteria capable of inducing growth and development of plants and protecting plants against phytopathogens [20]. PGPB increase plant tolerance to environmental stresses and facilitate plant growth through direct and indirect mechanisms.

##### 2.1.1.1 Direct mechanisms of PGPB

Direct mechanisms cause the balance of plant growth regulators [21].

• ***Expanding root growth***

It has been noticed that the PGPB could improve the absorption of water and nutrients by expanding and elongating the root system under drought and salinity stress [22]. In addition, other reports indicated that retaining higher water in inoculated *Triticum aestivum* L by PGPB may be due to the higher root biomass [23]. However, some studies indicated that PGPB can prevent the formation of root hair, leading to a reduction in plant development [24]. Reductions in root development of inoculated *Atriplex lentiformis* L. by PGPB can be related to the increase of reactive oxygen stress (ROS) in the cellular of roots [25].

• ***Fixation of atmospheric nitrogen***

PGPB inoculation is as a strategy of ecological and sustainable which increases plant growth and yield, however, it decreases the dependence of plants on N-fertilizer [26,27]. Some research has illustrated that plant growth and N-metabolism in inoculated *Zea mays* L. and *Sorghum bicolo*r L. were higher than in non-inoculated plants [28]. A study suggested that the increase in nitrogen concentration in *Solanum lycopersicum* L. was due to *Bacillus pumilus* inoculation [29]. The increase in N in inoculated plants by PGPB is probably related to the nitrogenase enzyme, which is encoded via nif genes such as nifH [30].

• ***Solubilization of mineral nutrients***

PGPB had an effective role in the solubilization of mineral nutrients (e.g., phosphate, potassium) in soil [31,32]. It has been reported that *Agrobacterium tumefaciens* was able to solubilize Zn in soil [33]. One of the reasons for the enhancement of mineral compounds solubilization in soil may be due to the production of siderophores through metabolites of bacterial [34]. In addition, a study showed that *Bacillus cereus*-Amazcala (B.c-A) increased the phosphate solubilization capacity [35].

• ***Production of phytohormones***

Some research has demonstrated that PGPB can be effective in increasing the production of phytohormones (e.g., auxins, cytokinins, and gibberellins) [36]. The production of some phytohormones, such as ABA, by PGPB can be due to aquaporin upregulation under stress conditions [37]. It has been reported that *Bacillus subtilis* can produce cytokinin and induce an enhancement in root and shoot weight [38]. In addition, *Bacillus amyloliquefaciens* produced a high concentration of gibberellic acid [35]. Inoculation of plants with PGPR increased plant growth. The production of growth regulators such as indole-acetic acid by PGPR seems to increase plant growth [39].

• ***Organic acids***

One of the important components of the rhizosphere is organic acids, such as lactic, oxalic, and citric acids [40]. They have an important role in rhizospheric bacterial chemotaxis and solubilization of phosphorus [41]. In addition, the PGPB has been documented to decrease pollutant levels and be useful for revitalizing organic pollutant-contaminated soils [42]. It has been reported that PGPB can induce the conversion of petroleum hydrocarbons, polychlorinated biphenyls, and pesticides into nontoxic products or minerals [42].

##### 2.1.1.2 Indirect mechanisms of PGPB

Indirect mechanisms are the biocontrol activities of PGPB to the biotic stress with antibiotics production, extracellular lytic enzymes, siderophore, and hydrogen cyanide [21].

• ***Antibiotic***

PGPB has shown that they can maintain genes for antibiotic and metal resistance [43]. In addition, some research has indicated that antibiotic resistance in plants can be a significant concern in the use of PGPB in agriculture [44]. Antibiotics of microbial substances such as agrocin 84, agrocin 434, 2,4-diacetylphloroglucinol, herbicolin, phenazine, oomycin, pyoluteorin, and pyrrolnitrin, siderophores can be effective on the reduction of pathogenic growth [45].

• ***Hydrolytic enzymes***

Some findings showed that PGPB are able to produce extracellular lysis enzymes such as chitinase, laminarinase, Q-1,3-glucanase, protease, and lipase and exhibit antifungal resistance against the harmful effects of plant pathogens [46]. The production of enzymes by PGPB can induce the transformation of organic material into stable materials via biochemical stages [44]. The results of the research showed that PGPB increased the extracellular lysis enzymes against nematode damage [47]. It has been reported that hydrolytic enzymes can lyse the cell walls of fungi, but not the cell walls of plants, and therefore hinder the expansion of phytopathogens [45].

• ***Siderophores production***

Organic compounds with low-molecular-weight and chemical ligands are named siderophores which highly tend to bind with iron [32]. Some studies found that siderophores-producing bacteria can reduce phytopathogens at the rhizosphere for the absorption of iron during root colonization [48]. It has been documented that PGPB increased the resistance of the plant to drought by increasing iron-chelating siderophores [49]. The unique significance of siderophores among the various microbial metabolites generated in the rhizosphere is related, on the one hand, to iron's crucial participation in essential plant metabolic processes and, on the other hand, to the element's unique characteristics in the soil. Additionally demonstrated is the function of PGPR strains in siderophore synthesis and plant pathogen management [50].

• ***Hydrogen cyanide***

A wide range of molecules with low weight such as hydrogen cyanide (HCN) are produced by PGPB against viral diseases [51]. It has been indicated that HCN inhibits the *Thielabiopsis basicola* pathogens in *Nicotiana rustica* L [45]. HCN-producing *Pseudomonas* strains were used for biological control of tomato bacterial disease, which led to disease reduction [52]. Also, in another study, HCN production by *P. fluorescens* strain CHA0 reduces the pathogenicity of fungi such as *Thielaviopsis basicola* as the cause of black rot in tobacco [53].

#### 2.1.2 Arbuscular mycorrhizal fungi

In agricultural and natural ecosystems, as biologically beneficial fungi, AMF can create an interaction of physical between plant roots and soils, which represent an essential part of agricultural ecosystems [54]. Nearly 90% of AMF can form symbioses with plant roots [55,56], contributing significantly to increasing plant uptake of macro and microelements in soils under environmental stress [57] and to improving soil density to create a protective barrier from pathogens and enhance water acquisition [14]. AMF can also protect crops against environmental stresses [58]. For instance, under drought stress, AMF may increase plant water uptake and turgor maintenance associated with osmotic balancing, and root hydraulic conductivity [58,59]. Overall, AMF play a beneficial role in producing metabolites such as essential oil [60], fatty acids [61], phytohormones [62], amino acids [63], antioxidant enzymes [16,59], and adjusting plant physiological statuses such as carbon dioxide exchange amount [64], stomatal conductance [65], photosynthetic pigments, proline content [66], and phenolic content [67]. It has been demonstrated that AMF can enhance photosynthesis activities and stomatal movement by developing the root systems [68,69]. Indeed, root colonization by mycorrhizal mycelium not only bolsters the root systems but also facilities the absorption of water and nutrient from larger soil volumes against drought stress [70]. Additionally, a raised nutrients uptake especially phosphorous by developing root system can provide the essential ATP and NADPH, which support oil and fatty acids biosynthesis [71]. Some researchers reported that AMF can decrease the accumulation of ROS by increasing flavonoids, carotenoids, anthocyanins, and phenols under water deficit [72].

Other beneficial microorganisms are *Trichoderma* [73] which can be applied as biological control generalists of plant diseases and pathogenic fungi with a well-shielded cropping system [74]. They can control pathogens by absorption of released nutrients (known as mycoparasitism) [75], production of antibiotics (e.g., aldehydes, alcohols, ketones, hydrogen cyanide, and heterocyclic nitrogen) [76], and generation of degrading enzymes (e.g., crystalline cellulose-hydrolyzing enzyme and b-glucosidase) in the cell wall [77]. *Trichoderma* species promote plant growth through a variety of processes, including the biological control of soil diseases through enzyme production and activity, and antibiotic synthesis [78]. *Trichoderma* species can colonize the rhizosphere at the critical “early germination” stage, contributing significantly to improving nutrient uptake and plant resistance to various stresses (e.g., heavy metal, salt, and drought stresses) [79] and they can serve as usual fungi of soil and rhizosphere to replace chemical seed treatment [77].

#### 2.1.3 Microbial consortia

Association between microorganisms and host plants can keep soil fertility and plant health, especially in low-input agriculture, which depends on biological prices than agrochemicals [80]. Indeed, in the microbial consortium, microbial species can perform synergistic interaction and give benefit each other. Some strains can maintain the non-producing strains against drought stress by producing secondary metabolites, such as exopolysaccharides [81]. A study showed that microorganisms belonging to the roots of grapevine and olive plants can improve the growth of *Orize sative* L. This enhancement may be due to the extensive roots system and increased water uptake ability [82,83]. In addition, it has been found that using humic acid and PGPR (*B.megaterium* and *B. subtilis*) enhanced the plant height and yield compared with untreated control. Indeed, Humic acid and PGPR enhanced the photosynthesis process by promoting stomatal conductance and stomatal density, thereby, improving the yield [84]. Also, it has been reported that the application of PGPB and N-fixing bacteria caused the improvement of root growth and resilience of plants against environmental stresses, as well as decreased N losses [85] PGPB can be used in the formation of ameliorating nodules in legumes when co-inoculated with rhizobia [14]. It has been found that *Bacillus polymyxa* and *Azospirillum brasilense* increased root colonization by *Glomus aggregatum*, and promoted biomass and phosphorus amount of palmarosa grass grew under irresoluble inorganic phosphate source [86].

### 2.2 PBM inoculation on plant growth

#### 2.2.1 Nutrients

Mixed or separate microorganisms can be inseminated within leaves, seeds, seedlings, roots, or soils. These inoculations cause the colonization of the rhizosphere or the inside of the plant, as well as, growth and toleration across abiotic stress stimulation [87]. PBM inoculation directly improves plant growth and productively, tolerance to abiotic stresses (e.g., drought, salt, and extreme temperatures) by increasing nutrient uptake, producing exopolysaccharides, osmoregulators, and antioxidants, regulating phytohormones (e.g., auxin, gibberellin, cytokinin, abscisic acid, and ethylene) [2] and/or indirectly protect plants against abiotic stresses by inducing systemic resistance, as well as producing siderophore and volatile metabolites [88]. Due to the increase in reactive oxygen species (ROS) production, peroxidation of lipids, free radical accumulation and elevated ethylene production, plant growth is inhibited during drought stress. Hence, the above events resulted in cell death and decreasing in photosynthetic rates and chlorophyll content. Also, PBM inoculation can positively affect germination indices of seed, seedling and early growth characteristics, root development and improve crop biomass and productivity [89,90].

It has been proved that PBM can be used as biofertilizers to increase the stock of macro and micro-elements, boost plant growth and decrease the application of chemical fertilization [57]. Since the essential nutrients for plants mainly include nitrogen, phosphorus, and iron, among PBM selection tests, nitrogen fixation, phosphate solubilization, and siderophore production are widely investigated [87]. One of the essential macro-elements for synthesizing proteins and nucleic acids is nitrogen. It has been reported that PGPB strains such as *Azospirillum, Azotobacter, Achromobacter, Rhizobium* and *Klebsiella* can fix biological nitrogen via decreasing nitrogen gas (N_2_) to ammonia (NH_3_) [91]. Moreover, phosphorus is an urgent plant nutrient for growth, which participates as a structural ingredient of nucleic acids, phospholipids, and adenosine triphosphate (ATP) [92,93]. Some PGPB strains such as *Rhizobium, Bacillus, Pseudomonas, Azotobacter*, and *Azospirillium* can dissolve phosphate and convert insoluble organic and inorganic phosphate into available plant form, which are called phosphate-solubilizing bacteria (PSB) [94]. Organic acids (gluconic or keto-gluconic acids) produced by PSB along with their carboxyl and hydroxyl ions chelate cations and reduce pH to release phosphorus [95]. Furthermore, PGPB act a main role in metabolic and biochemical pathways, especially for biological nitrogen fixation and photosynthesis [96]. It is known that large proportions of soil-phosphorus remain interlocked in various insoluble forms and are unavailable for plants. PBM can decrease soil pH through execration of organic acids such as gluconate, citrate, lactate, and succinate that leads to the acidification of the surroundings and microbial cells, therefore, phosphorus ions are released by substitution of *H*^+^ for Ca^2+^ [97]. In addition, iron is one of the essential micro-elements for the biosynthesis of chlorophyll, photosynthesis, and respiration. As a chelator, siderophores have a great specificity to bind iron, continued by the transport and deposit of Fe^3+^ in bacterial cells [98]. *Burkholderia, Enterobacter, Grimontella*, and *Pseudomonas* can be used as siderophore producers to promote plant nutrition and inhibit phytopathogens via sequestration of free environmental iron [91].

#### 2.2.2 Phytohormones

Phytohormones are organic compounds that are responsible for plant development. PBM can modulate phytohormones, such as auxins, cytokinins, gibberellins, abscisic acid, ethylene, salicylic acid, brassinosteroids, jasmonates, polyamines, and strigolactones [99]. A study reported the increased auxin and gibberellin in leaves of *Zea mays* inoculated by PGPB [100]. The negative effects of drought, chilling, heat, or salinity stress can be alleviated by PBM inoculation via auxin production, gibberellin, cytokinin, ACC deaminase, abscisic acid strigolactones, and jasmonates [101]. It has been demonstrated that PBM inoculation increased auxin concentration in plants and improved the growth of various plant species (e.g., *Zea mays, Brassica juncia, Fagopyrum esculentum*, and *Saccharum officinarum*) by improving uptake of water and nutrient [102]. The auxin produced by PBM is a beneficial phytohormone that regulates cell division [103]. PBM can improve plant-related parameters (e.g., seed germination, development of leaves, stem, flower and fruit) by enhancing gibberellin [104]. Under saline conditions, PBM inoculation can increase the concentrations of abscisic acid, jasmonates, and brassinosteroids in plants [105].

#### 2.2.3 Exopolysaccharides

Microorganisms can form a productive biofilm on the root surface by producing exopolysaccharides [106]. In this way, this mechanism causes the increase of water keeping in soil particles and maintains soil moisture in the rhizosphere. In addition*, Streptococcus epidermidis* can protect the cells of plant roots against osmotic stress and enhance environmental stress tolerance [106]. It was suggested that *Pseudomonas putida* strain GAP-P45 as an exopolysaccharide producing bacterium can cause the biofilm formation on the root surface in *Helianthus annuus* seedlings and increase tolerance of seedlings against drought stress [107]. In addition, other studies have demonstrated that proline accumulation, sugars and free amino acids increased in plants inoculation by exopolysaccharides producing bacterium *Pseudomonas aeruginosa* and *Azospirillum* spp. under drought stress [[108], [109], [110]].

#### 2.2.4 Antioxidants

The PBM can enhance antioxidant enzymes activities such as ascorbate peroxidase (APX), catalase (CAT) and superoxide dismutase (SOD), and antioxidant non-enzymes such as glutathione (GSH), carotenoids, tocopherols, and phenolics to alleviate ROS accumulations that are caused by various stresses [32,102]. Increased activity of CAT and APX due to inoculation of *Cuminum cyminum* seeds with *Pseudomonas fluorescens* and *Trichoderma harzianum* under drought stress conditions has been reported [16]. *Linum usitatissimum* inoculation with *P. fluorescens* enhanced antioxidant enzymes such as CAT, APX, and GSH in storage conditions [111].

#### 2.2.5 Osmoregulants

Against drought and salinity stresses, microbial inoculants can produce osmoregulants such as carbohydrates, proteins, amino acids lipids, proline, glycine betaine, and trehalose [112]. Osmoregulants induce the stabilization of protein and membrane structure under dehydration conditions, maintain osmotic balance across the membrane, and ensure protein correct folding under salinity stress [90]. It has been found that *Burkholderia phytofirmans*. can increase plant tolerance across low temperatures by modifying carbohydrate metabolism [113]. Also, *Pseudomonas fluorescens* has been found to promote plant tolerance against water stress by enhancing catalase and peroxidase enzyme activities and proline accumulations [114].

### 2.3 Inoculation methods of PBM

**Fig. 1:**
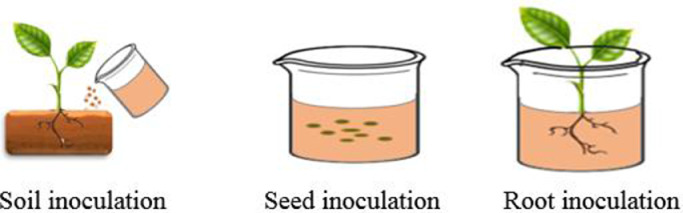
Methods of inoculation of PBM in host plan.

Different methods of PBM inoculation on host plants can affect the survival and reproduction of microbes crowded into the rhizosphere and their ability to promote plant growth [115]. Due to the fact that the mobility of microorganisms in the soil is low, microbial inoculants should be placed in the vicinity of the rhizosphere. To spread microbial inoculants around the rhizosphere, nematodes can be used as a vector for their inoculation [116]. Except for inoculant density and methods of inoculation, the response of the plant to PBM inoculation and their colonization is also important for microbial functioning [117]. After inoculation, the reduction of microbial population in the rhizosphere may be due to unadapted microorganisms to their new environment. However, root exudations play a critical role in microbial growth. Besides, biotic and abiotic factors can also affect the functional variety of microbial populations [115]. Microbial inoculation can be carried out with a single isolate or microbial consortia (e.g., co-inoculation). It was found that co-inoculation improves the efficiency of inoculation and plant development [87]. Different methods including seed, soil, root, and foliar inoculation are used to inoculate plants with PBM (Fig. 1; Table 1).

**Table 1:** Effects of different methods of PBM inoculation on plant growth.

PBM	Plant	Inoculation	Effect inoculation	References
*Burkholderia phytofirmans*	*Ryegrass*	Seed, soil, root	Soil inoculation increased plant biomass production	[118]
*Pseudomonas* sp.	*Cicer areietinum*	Seed, soil	Method of soil inoculation was more effective on plant growth than seed inoculation	[119]
*Rhizobia*	*Oryza sativa*	Seed	Seed inoculation was effective in increasing plant growth	[120]
*Streptomyces, Aspergillus, Bacillus*	*Triticum* sp.	Seed	Seed inoculation increased the yield and reduced the Rhizoctonia root rot	[121]
*Pseudomonas aeruginosa, Bacillus amyloliquefaciens, and Trichoderma harzianum*	*Oryza sativa*	Soil	Soil inoculation could manage disease of aerobic rice	[122]
*Pseudomonas fluorescens*	*Arabidopsis thaliana*	Root	Root inoculation protected leaves against the oomycete *Peronospora parasitica*	[123]
*Pseudomonas putida*	*Zea mays*	Root	Root inoculation reduced leaf necrosis	[124]
*Bacillus megaterium, Trichoderma longibrachiatum* and *Trichoderma simmonsii*	*Glycine* max	Seed	Seed inoculation improved germination and seedling indices against cold stress	[125]
*Providencia rettgeri, Advenella incenata, Acinetobacter calcoaceticus,* and *Serratia plymuthica*	*Avena sativa, Medicago sativa* and *Cucumis sativus*	Soil	Soil inoculation increased the available nutrient (e.g., N, P, K) content	[126]
*Bacillus subtili*	*Triticum aestivum*	Soil	Soil inoculation decreased the chromium toxicity	[127]

The seed inoculation technique is the most applied method [128]. Advantages and disadvantages of each inoculation method depend on the tool accessibility, inoculum and seed type (e.g., size, shape, and fragility), the presence of inhibitory components in the seed (e.g., fungicides, micronutrients, and PBM), and costs (Table 2).

**Table 2:** Methods of PBM inoculation.

Method	Technique	Advantage	Disadvantage	References
Soil inoculation	Granular/powder; liquid inoculation; immobilized microbial cells	Prevents damage to seeds and cotyledons/ Reduce the destructive effects of fungicides and pesticides	Needs specialized tools for using and larger rates of inoculants; needs s more storage area and transportation; costly method	[129]
Root inoculation	Foliar spray; root dipping	Microbial inoculation of seeds in high concentration	It requires a lot of microbial inoculation and is time consuming and expensive	(Fernandez et al. [120])
Seed inoculation	Seed bio-priming and Seed coating	Cost-effective, accurate and useful for improving the properties of seeds	Reduction of microbial inoculation survival	[130, 16]

#### 2.3.1 Soil inoculation

The method of soil inoculation is the direct transmission of PBM to the soil via drenching, soil incorporation, and microcapsules [129]. Inoculation of *Brachiaria brizantha* seeds by *Burkholderia pyrrocinia* and *Pseudomonas fluorescent* was not successful, in contrast, the soil inoculation with drenching improved plant growth and seedlings emergence [87]. Soil inoculation with *Pseudomonas* sp. resulted in better nodulation and growth than seed inoculation of *Cicer arietinum* [119]. Recently, it has been found that soil inoculation with PGPB improved the growth, the productivity of nutrient and water uptaking by roots of *Ranunculus asiaticus* [131]. It has been shown that direct soil inoculation with PGPB and AMF boosts growth, yield, and nutrient uptake [132]. Soil inoculation with *Pseudomonas aeruginosa, Corynebacterium agropyri*, and *Enterobacter gergoviae* was more significant on the disease suppression of aerobic rice compared to *Bacillus amyloliquefaciens, Trichoderma harzianum* and *Trichoderma virens* [122]. A study suggested that nutrient availability increased after soil inoculation of *Providencia rettgeri, Acinetobacter calcoaceticus* and *Serratia plymuthica* [126]. Soil inoculation using *Bacillus subtilis* has been reported to decrease the toxicity of chromium in *Triticum aestivum* [127].

#### 2.3.2 Root inoculation

In this method, the roots immerse in a microbial solution [129]. After microbial inoculation, the seedlings are grown at a proper substratum for their development. In this way, this method provides plant size standardization and also causes the direct relationship between roots and inoculants to improve root colonization [133]. The inoculation of *Burkholderia phytofirmans* with *Vitis vinifera* roots plant's low-temperature tolerance, altered carbohydrate metabolism, and improved plant growth and yield (Fernandez et al. 2012). Root inoculation of *Oryza sativa* with *Rhizobia* was more efficient in improving plant length compared with seed inoculation [120]. One study found that root inoculation with *Pseudomonas fluorescens* caused an increase in induced systemic resistance in leaves of *Arabidopsis thaliana* [123]. The inoculation of *Pseudomonas putida* with roots of *Z. mays* caused the reduction of leaf necrosis [124].

#### 2.3.3 Seed inoculation

To decrease the use of chemical seed treatment, the method of seed inoculation with PBM is a better alternative. In this method, seeds immerse in the microbial solution of known concentration. During the germination process, the seed releases carbohydrates and amino acids in the exudates. In turn, microorganisms use the released seed exudates as the nutritional source in soils and then colonize plant roots [130]. It has been reported that the inoculation of *Burkholderia phytofirmans* with *Ryegrass* seeds enhanced plant growth, hydrocarbon degradation, and phytoremediation [118]. Association of PBM with plant roots caused the modulated phytohormones levels. Compared with seedling inoculation, seed inoculation with PGPB and AMF has been more effective, stimulating the growth and wood production of *Schizolobium parahyba* var. *amazonicum* [134]. While the growing root tips have not been activated, inoculum stays dormant in the soil [135]. In a study, inoculation of wheat seeds of *Streptomyces, Aspergillus, Bacillus* with seeds of *T. aestivum* caused the increased grain yield [121]. Under cold stress, the inoculation of *Glycine* max seeds with *Bacillus megaterium, Trichoderma longibrachiatum* and *Trichoderma simmonsii* was more efficient in increasing germination indices and seedling growth [125].

### 2.4 Mechanisms PBM to survive in diverse conditions

Microorganisms can induce several mechanisms to cope with stressful conditions and improve the growth of host plants. Some microbes survive under low and high temperatures, drought, salinity, acid and alkaline conditions [87] through modification of cell walls, metabolic responses, and gene expression [90]. Some microorganisms (e.g., *Bacillus* sp., *Azospirillum* sp., and *Pseudomonas* sp.) can secrete volatile organic compounds (VOC) (such as alkyl sulfides, indole, and terpenes). The signal interactions between plants and microbes can be achieved through the distribution of VOC in soil pores [136]. Microbes can accumulate amino acids and avoid dehydration and death against low soil moisture [117]. AMF increased soil organic carbon and changed the microbial population in the rhizosphere, thus causing the modification of the rhizosphere [137] The pigments produced by *Bacillus* and *Serratia* can clear radiation and stop DNA damage against high light [138]. Microorganisms such as *Azospirillum* sp.*, Pseudomonas* sp., and *Bacillus* sp. significantly influenced soil micronutrient accessibility through reduction of solubilization, chelation and oxidation, and altered the pH of their surrounding soils [91].

### 2.5 Influence of abiotic factors on PBM

**Fig. 2:**
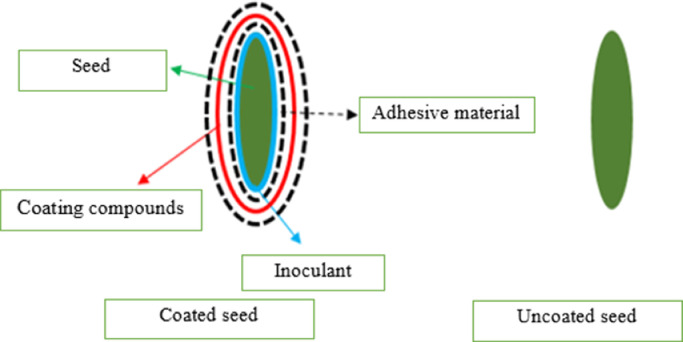
Schematic of coated seeds (left) and uncoated seeds (right).

The abiotic factors can induce stress in the metabolism of plants and modified the compositions of root exudates. This can affect the microbiome in the rhizosphere and the interactions between plants and microbes. In this way, the benefits of PBM can be declined by abiotic factors (Fig. 2).

#### 2.5.1 Soil

Soil pH is an important factor in influencing the solubility of various metallic ions and the accessibility of nutrients, as well as the physical properties of the soil. One of the problems with agricultural productivity in the world is high or low pH. Soil salinity can limit plant growth and thus crop productivity. Hence, these conditions reduce the nutrient deficiency and yield and cause ion toxification, osmotic and oxidative stress [139]. Salinity stress influences crop production by declining the levels of mineral availability and growth regulators, and persuading ions interceded toxicity, osmotic stress, and ROS production, which conclusively causes the blockage of seed germination, seedling growth, the onset of flowering and fruit [140]. The conditions of soil nutrition also influence PBM efficiency. It has been evaluated that inoculation of PBM was more effective on growth in nutrient-poor conditions [115]. Inoculations of *Pseudomonas* sp., *Bacillus* sp., and *Mycobacterium* sp. caused enhanced plant growth in soils with a nutrient deficit [141]. In addition, heavy metal contamination in soils can inhibit the beneficial effects of inoculants on plant growth and agricultural productivity [141]. However, *Pseudomonas aeruginosa, Alcaligenes feacalis*, and *Bacillus subtilis* can serve as an effective remedial approach to increase plant tolerance against heavy metals [142]. Another research revealed that *Klebsiella variicola* and *Azospirillum* sp. caused the improved growth and tolerance of *Glycin* max [143] and *Z. mays* [144] under flooding stress.

#### 2.5.2 Water

PBM such as *Azotobacter chroococcum* and *Azospirillum brasilense* in *Mentha pulegium* [145], *Pseudomonas* sp. and *Azotobacter* sp. in *Cymbopogon citratus* [146] can promote plant tolerance against drought stress. Increased soil temperature by water stress can inhibit PBM multiplication. In addition, it has been reported that flooding condition causes the reduction of O_2_ availability in the soil and restricts the aerobic respiration of microorganisms in soils [147]. The type of microorganism and light intensity can influence PBM efficiency [135].

#### 2.5.3 Light

Light may alter the interactions between plants and micro-organisms by changing the quantity and chemical compound of root exudates [135]. The colonization of microorganisms depends on plant-provided carbohydrates in exchange for nutrients. Under limited light intensity, inoculation of PBM such as *Kaistobacter* sp. and *Pseudomonas* sp. can enhance the growth of *Ophiopogon japonicus* and *Lolium perenne* [148]. The microbial root symbioses such as *Paraglomus* sp., *Rhizophagus* sp., and *Rhizobium* inhibited the growth of *Phaseolus lunatus* [149].

#### 2.5.4 Temperature

Temperature can interfere with interactions between plants and microorganisms by changing root exudation composition, as well as, affecting the morphological, biochemical, and physiological attributes of plants [150]. Inoculation of PBM such as *Pseudomonas putida* and *Bacillus cereus* can increase the growth of *Triticum* sp. and *Solanum lycopersicum* and decrease the negative effects of high-temperature stress [151]. Similarly, it has been found that the inoculation of *Burkholderia* sp. increased the tolerance and yield of *Vitis vinifera* under low temperatures (Fernandez et al. 2012).

## 3 Microbial seed coating?

### 3.1 Definition of PBM seed coating

**Fig. 3:**
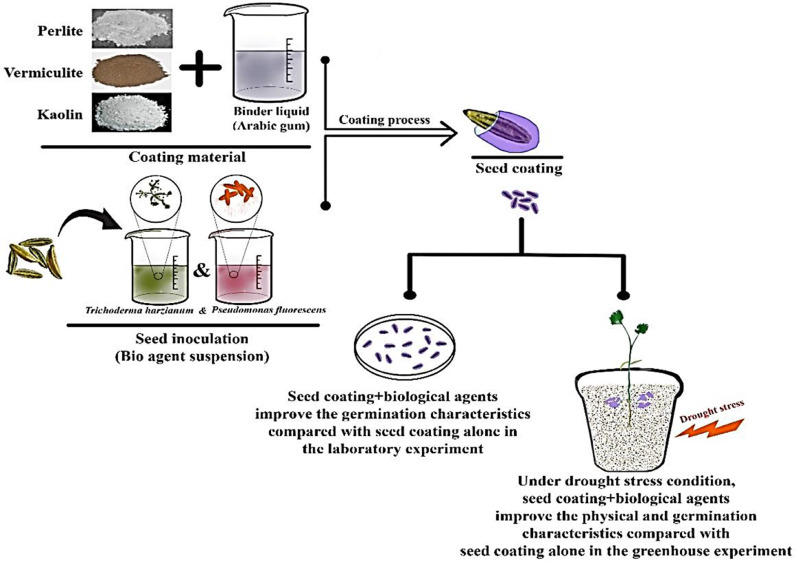
The impact of biological agents and fillers seed coating in improving drought stress tolerance of plants [155].

In the last years, the application of microorganisms as alternatives to chemical treatments in agricultural products and pastures has increased against various stresses [152]. Seed coating is the application of exogenous onto the seed external to boost seed form and handle characteristics such as seed size and weight and delivery of energetic compounds (e.g., plant growth regulators, micronutrients, and microbial inoculants), consequently protecting the seeds from phytopathogens and enhancing germination and plant growth [153]. It is well established from a variety of studies that seed coating with PGPB (such as *Pseudomonas* sp., *Bacillus* sp.), AMF, and *Trichoderma* was an effective and suitable strategy that could introduce PBM into the rhizosphere and provide them to plant roots and other tissues [20]. In comparison to traditional seed treatments, seed coating for different crops was a promising tool that causes a reduced use of inoculum [17]. Seed coating with PBM could protect plants against pathogens and improve seed germination against environmental stresses (e.g., drought and salinity) and agrochemicals (e.g., pesticides, growth regulators, and mineral fertilizers) [[14], [154],]. Generally, different equipment and methods are used in seed coating to attain good application uniformity and adherence. The use of appropriate seed coating equipment and methods stand can improve plant establishment and seedling vigor under environmental stresses [154,153]. In both optimal and drought-stress conditions, seed coating with the strains of *Trichoderma harzianum* fungus and *Pseudomonas fluorescent* bacteria and fillers treatment improved the physical qualities of anise seeds while also enhancing the early vegetative growth of anise seedlings under greenhouse conditions [155] ((Fig. 3).

### 3.2 Ingredients of seed coating

The materials used in seed coating include a binder, filler, carriers, and active ingredients (Fig. 2), which assist to release a suitable amount of PBM in physiologic conditions [154]. Binders are polymers such as the natural and syntactic origin, which ensure the adherence and cohesion of the material on the seed surface and keep the ingredients active [17]. The Arabic and xanthan gum can be applied as binders to develop the survival of bacteria, rhizobia, and AMF applied to seeds [156].

The fillers are generally static powders (such as bentonite, calcium carbonate, talc, diatomaceous earth, sand, and wood dust), which can be single or mixed to modify seed shape, size, and weight [18,154,153]. Nowadays, biochar and chitosan are used as fillers in microbial seed coating [157]. In seed coating, the carriers as one of the factors affecting inoculants should be compatible with these materials and also have the ability to retain sufficient moisture for the growth and survival of inoculants [156]. In seeds coated with bio-treatments, some materials such as vermiculite, perlite, etc., are used as carriers, which have high water holding capacity, non-toxicity to seeds, and the capability to stick to the seed external [154] and also can assure seedling emergence and quality and the survival of PBM on the seeds [156].

Active ingredients are different from those used in processes of seed coating. The most common active ingredient is protectants, including fungicides, pesticides, insecticides, nematicides, predator deterrents, and herbicides [158], which is used to promote germination and emergence, growth and yield by decreasing predation and putridity by pathogens [159]. However, sometimes these protectants can negatively affect the germination rate [160]. Nutrient amendments, namely macronutrient (e.g., N, P, and K) and micronutrients (Bo, Cu, Mn, Mo, and Zn) applications in seed coating positively affect germination, growth and yield of plants, and also reduce the negative effects of osmotic stress [153]. The most common application of symbiotic microorganisms into coatings involves the inoculation of rhizobia for legumes. The rhizobia-friendly coating formulizations along with the election of desiccation-resistant bacteria modified the survival of symbiotic microorganisms and the beneficial storage life [161]. To attract and hold water close to the seed, soil hydrophilic materials have been extensively used in seed coating. In addition, the soil surfactant applies within the seed coating materials to enhance water availability to seeds and seedlings in water-repellent soil [162].

A range of components (such as PBM, amino acid, chitosan, and soy flour) can be used in the processes of coating seeds of crop and vegetable species in order to stimulate germination and growth, improve stress resistance and establishment, disease reduction, restoration efficacy of native seeds, and protect the finite resource and enhance business for seed technology [153]. The incorporation of fluorescent colorants and magnetic powder into coatings has been expanded to meliorate the traceability of seed batches via the supply chain [153].

### 3.3 Machines of seed coating

In general, three major kinds of seed coating tools containing a fluidized bed, rotary coater, and rotary pan are used to procreate five kinds of seed coatings, namely dry coating, seed dressing, film coat, entrustment, and seed pellet.

The rotating pan was the first device applied for seed coating, consisting of a circular and usually sloping container rotated by a motor. The seeds were placed in a pan, while the container was spinning, liquids were sprayed on the seeds with a nozzle, and powders were added via a blower or hand spraying [17,153]. The round pan is widely used in the different seed coating methods [[86], [163]].

The fluidized or spouted bed apparatus is a cylindrical apparatus that causes the rotation of seeds by airflow through the spray nozzle that atomizes the coating liquid towards the suspended seed mass. This process is used for film coating and surface incrustation, but it is not possible for pelleting.

The rotary coater is an apparatus used in the pelleting and film coating, and it includes a cylindrical drum with a concave disk at the base. Its rotation leads the seed mass to whirl in a regular flow along the drum wall. Usually, a smaller rotating disk attaches to the drum lid and suspends in the middle of the drum. It is accountable for atomizing and spraying [153]. In the seed coating industry, these systems are standard in seed treatment. However, nowadays, considering seed coating commercialization and industrial, a lot of information is not disclosed regarding tools and details of seed coating methods.

### 3.4 Types of seed coating

#### 3.4.1 Dry powder coating

Dry powder coating is a method in which seeds are placed in a dry powder and mixed. Also, dry powder can be utilized for bacterial or fungal treatments followed by drying (hydration/dehydration) [17]. There is a rotating brush made of stainless steel which sieves a powder material using a dosing sieve [118]. It has been reported that talc and graphite are the most common dry powders [164]. The dosage for dry coating powders used onto seeds is extended with their adherence to seeds and ranges from 0.06 to 1.0% of seed weight [17].

#### 3.4.2 Seed dressing

Seed dressing coating is a method that uses a low dosage of active ingredients to create a thin layer around the seed. In this method, the active materials especially chemical protectants can be used in a wide range [165]. The most common equipment in seed dressing is the rotary coater. The rotary coater places the liquids onto a spinning disk and atomizes onto seeds that are spinning inside a metal cylinder, then discharges the freshly treated seeds. The dosage of liquid seed treatment formulations typically ranges from <0.05 to 1.0% by weight [17].

#### 3.4.3 Film coating

**Fig. 4:**
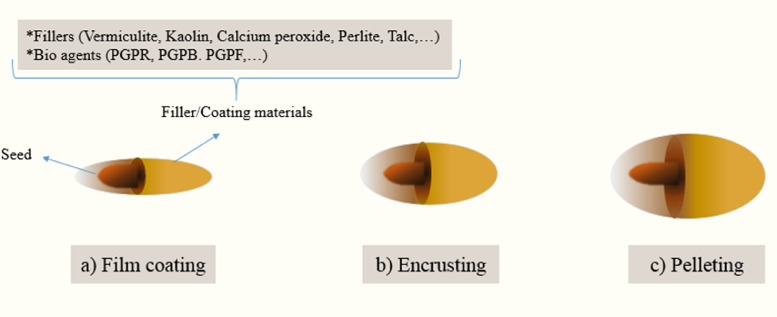
The three major types of seed coating: film coating (a), entrusting (b) and pelleting (c).

Film coating is modeled based on the industries of pharmaceutics and confectionary [17] (Fig. 4a). In this method, the seed size does not change, and a small layer (less than 10% of the seed weight) of coating materials such as pigments, fungicides, and polymers are placed around the seed [154]. During this process, the shape and size of the seed do not change and its application creates successful sowing in the field and protects the environment [17]. Nowadays, film coating has been considered an effective and reliable tool to improve crop productivity in the seed industry.

#### 3.4.4 Encrusting

Encrusting is the process of coating seeds by adding liquids and solid particulates to complete seed coverage (Fig. 4b). In this method, the original seed shape is retained, and seedling emergence is improved [17,153].

#### 3.4.5 Pelleting

In the seed pelleting process, seeds are coated with inert materials (such as vermiculite, kaolin, calcium peroxide, perlite, talc, and diatomaceous earth) so that the initial size or shape of seeds is not clear (Fig. 4c). Ultimately, seed pelleting cab changes thin seeds into larger and spherical-shaped ones, which could help cultivate very small with low vigor seeds [17,153].

According to these characteristics of the natural coating of seeds, mainly different agents are used for seed coating, such as protectants, micronutrients [166], microorganisms (bacteria and fungi) [20]. The uptake and translocation of agent compounds into seeds can be performed through imbibing seeds in water or emerging radicle and root systems. Overall, the applications of agents are effective in improving seed germination rate, establishment, and increasing the yield of crops [154,153].

### 3.5 Formulation process of seed coating

Three basic components, namely the elected microorganism, an appropriate carrier (solid or liquid), and various additives can be applied to create an efficient formulation of PBM [14]. Various factors such as incorrect formulation of the inoculant and limited shelf life may preclude the application of seed coating [167]. The formulation acts a significant role in the inoculation process as it can determine the bioagent potential [156]. Nowadays, formulation development by industries is essential to commercialize biocontrol technologies. The expansion of optimal formulations with appropriate carriers for the utilization of microbial inoculants contributes significantly to the control and management of pathogens and seed-borne diseases in crops [168]. There are several types of formulations such as wettable powder, liquid, and granular used in soils or spray applications [169].

### 3.6 Shelf life of the microbial coating

An essential commercial issue for seed coating is microbial survival [170]. Several factors such as coating type, inoculants (e.g., strain, type, purity, sterile or not, moisture status, and age), coating carrier (e.g., silica, carboxymethyl cellulose, and biochar), drying process (e.g., polymer, final moisture status, time, and temperature), storage condition (e.g., temperature, humidity, water status, polymer, and contaminants) can affect the survival of microorganisms [154]. It has been reported that the changes in physiology and morphology of cells during inoculants can influence the survival of microorganisms physiological and morphological changes of cells during inoculants can influence the survival of microorganisms [171]. In structural biopolymers, water activity and its solvent properties can inhibit the survival of microorganisms during desiccation [172]. Also, at different relative humidity, polymers influence water available to microorganisms by moisture sorption [173]. One of the most important factors influencing rhizobia survival on seeds is desiccation [152]. During inoculation and inoculated seed storage, the expansion and rate of desiccation depend on the ambient relative humidity. For instance, some studies have reported that relative humidity and water activity are effective in the survival of rhizobia [174]. Many evidence demonstrated that the survival of microbes is improved when the difference in water status between intracellular and extracellular is reduced [175]. Low relative humidity storage of the environment may increase the survival of freeze-dried cells or decrease the survival of completely hydrated survival cells [[154], [176]]. For the survival of cells, rehydration is important to improve the cell viability of microbes by decreasing water influx via cell membranes [152,154]. The polymeric adhesives or coating materials include pigments, nutrients, and protection agents of seeds that can be applied to enhance the survival of microbes on seeds [152,173]. Furthermore, polymers can increase the ability to protect cells of microbes against different environmental stresses [154]. It has been indicated that drying seeds of *Trifolium subterraneum, Trifolium repens*, and *Medicago sativa* after coating can enhance microbial survival for a long time [152].

### 3.7 Delivery methods

Innovative seed coating technology can provide the delivery of many kinds of materials that are effective in the enhancement of seedling establishment and plant growth [156]. Some studies have shown that several bacteria, including *Pseudomonads fluorescent, Pantoea* sp., *Bacillus cereu,* and the fungus *Trichoderma harzianum* played important role in controlling a range of soil-borne diseases [177]. Seed bio-priming is a proceeding of coating seeds with fungal or bacterial agents in which biological and physiological treatments are used to control the disease [178]. Coating rice seeds with two biological agents *Pseudomonas* and *Bacillus* could protect rice against *Xanthomonas oryzae* and increase seed quality and germination [179]. Using *Pseudomonas fluresences* SP700s bacteria as the coating factor not only increased emergence percentage and yield of rice but also reduced dirty panicle disease incidence and severity [180]. The *Trichoderma atroviride* inoculated corn seeds had the highest percentage of germination [181]. The pathogens of seed-born and soil-born can form a host-parasite relationship through the root. In this regard, PBM can protect the rhizosphere zone against soil-borne diseases. It was demonstrated that the inoculation *Trichoderma harzianum* in soil was more effective in controlling *Armillaria root* rot in *Camellia sinensis* [182]. The inoculation of the combinations of *Pseudomonas fluresences* and *Bacillus subtilis* could prevent the growth of pathogens on the wheat roots [177]. A study investigated the influence of *Bacillus subtilis* and *Pseudomonas fluorescent* on the germination indices and seedling growth of *Cuminum cyminum* under salinity conditions. Results from this study demonstrated that bacterial inoculation improved the germination and seedling characteristics in both optimal conditions and salinity stress [183]. However, the co-inoculation of *Bacillus subtilis* and *Pseudomonas fluorescent* caused a decreasing in plant growth and yield. According to some results of research [184], inoculation of single *Rhizophagus irregularis* or dual *Pseudomonas libanensis* + *Rhizophagus irregularis* under greenhouse did not affect cowpea seed yield, however, application of *P. libanensis* increased plant growth performance. Similarly, co-inoculation of *Trichoderma* sp., *B. bassiana, Metarhizium anisopliae*, and AM fungi had no effect on seed germination of *Lactuca sativa* [185]. A reserch showed that coating of *Triticum turgidum* seeds with *P. fluorescens* was more effective on growth parameters than *B. subtilis* and *F. graminearum* [177]. A study on the evaluation of biological control of wheat root in field conditions reported that using *P. fluorescents* was the most effective treatment compared to other treatments [186].

### 3.8 Application of microbial seed coating in the agricultural system

For billions of years, it has been proved that microorganisms had an intense influence on the whole planet [187]. Nowadays, the enormous diversity of microbes and their ability on the earth have been known [188]. For instance, bacteria and fungi can manage agricultural sustainability in the world [187]. It has been confirmed that for developing a sustainable strategy, the application of microbial seed coating in crop production systems can increase crop production, improve resource use efficiency, and protect plants against phytopathogens [189].

#### 3.8.1 Enhancement of plant growth and yield

Standardization of size, weight, shape, and uniformity of seeds in seed coating can enhance plantability in the field and crop growth and yield [154]. While morphological characteristics of the seeds are improved by seed coating, however, seed coating may be an obstacle to germination and emergency [177,183]. A study has pointed out that delayed germination of *D. carota* [190], and *Z. mays* [191] caused by seed coating is due to coating combinations on imbibition of water and available oxygen. To increase the longevity of coated seeds and microbial functionality in situ, the application of an effective formulation plays a role in the expansion of commercial coated seeds [[14], [154]]. Application of polymeric adhesives (such as polyvinylpyrrolidone, xanthan gum, methylcellulose) could maintain water activity levels optimal in coating formulations to improve the viability of inoculants [173]. To achieve food security and sustainable agriculture, seed quality such as germination, vigor, and mister content is important. Therefore, the microbial seed coating is the seed's primary defense from unfavorable environmental conditions and pathogens, thus improving seed viability and vigor [179]. The impact of PBM on plant growth has been reported for numerous crops grown in greenhouse and field environments [181].

Using PBM in seed coating can enhance the percentage of germination, seedling indices, and subsequent plant growth in both optimum and stress conditions [[14], [154]]. It has been reported that the yield and macro and microelements, antioxidant activity, total phenolic, caffeoylquinic acids, and flavonoids increased in propagated *Cynara cardunculus* seeds coated by *Rhizophagus intraradices, Funneliformis mosseae,* and *Trichoderma atrovirid* [192]. It has been determined that the use of *Pseudomonas fluorescence bacteria* and *Trichoderma harzianum* in the coating of *C. cyminum* seeds improved seedling emergence rate and seedling growth, antioxidant activity under drought stress in greenhouse conditions [16]. Similarly, it has been shown that seed coating of *T. turgidum* with *Rhizophagus irregularis* BEG140 using silicon dioxide resulted in an enhancement in shoot dry weight, seed weight and nutrition (K and Zn) contents under low fertilization. Some entomopathogenic fungi associated with plant roots can protect the host plants against disease and insect pests [193]. For instance, the seed coated with entomopathogenic fungi such as *Metarhizium* and *Beauveria* protected *Z. mays* against *Costelytra giveni* and *Fusarium graminearum* and improved germination and growth [194]. Seed coating through the formulation of *T. harzianum, T. viride* and *T. atroviride* enhanced plant growth and germination of *Z. mays* var. saccharata, *T. aestivum*, and *Beta vulgaris*) [195]. In addition, it has been reported that the use of *Methylorubrum aminovorans* in the coating process of *Arachis hypogaea's* seeds caused an increase in germination and growth [196].

#### 3.8.2 Alleviation of abiotic stress

The use of PBM as biocontrol agents is an attractive management strategy for both the conventional and organic farming industry that can meliorate plant growth and performance under optimal and stressful conditions and also defend plants across a diversity of soil and seed pathogens [197]. Besides, several factors have an effective role in the success of microbial seed coating for biocontrol purposes including cultivation practices, dosage, timing, and method of PBM application [4]. Environmental stresses such as biotic stresses (e.g., drought, salinity, extreme temperatures, and nutrient deficiency, etc.) and abiotic stress (living organisms such as bacteria, viruses, parasitic nematodes, insects, weeds, and other indigenous) are environmental factors that may limit worldwide crop production [[153], [154]]. It has been suggested that several PBM (bacterial and fungal strains) such as *PaeniBacillus alvei* and *Bacillus amiloliquefaciens* in potato, *Pseudomonas* sp. in potato and strawberry, and *Talaromyces flavus* in tomato were successfully protected plants against *Verticillium dahliae* [198]. The bacteria and fungi application had affirmative agents on plant growth against drought stress and facilitated plant growth and development by supplying mineral nutrients and phytohormones [199]. In a greenhouse, research was found that seed coating of the combination of microbial strains, polymers with several doses of trace and macro-micro-nutrients with *Z. mays, G.* max, *Brassica napus, T. turgidum, Hordeum vulgare*, and *Lens culinaris* under water-stressed conditions helped to fix plant cell membranes and decreased the damages from drying cycles, and eventually enhanced crop productivity under water stress [200]. Seed coating of *Vigna unguiculata* with *Bacillus* sp. could improve the growth and production, and nutrients of crops and decreased usage of the chemical fertilizers in arid agriculture [201]. The use of a combination of genus *Pseudomonas, Azotobacter, Azospirillium* and *Rhizobium* as biofertilizers in coating materials of cotton seed enhanced the growth, relative water content, and contents of chlorophyll and ionic (*K*^+^/Na^+^) under both salinity and normal conditions, but decreased shoot growth and leaf gas exchange under salinity stress [202]. In an experiment performed under salinity stress conditions, coating maize seeds with *Bacillus* and *Pseudomonas*, and *Pseudomonas* produced more IAA and ACC deaminase, different hydrolytic enzymes, and antifungal activity against two fungal pathogens compared to non-salinity stress [203]. Co-inoculants of AMF and PGPB onto seeds of soybean in the laboratory and under greenhouse conditions improved the germination, seedling growth, and potassium uptake under drought and salinity stress [125]. In the greenhouse experiment, the growth and photosynthetic state of *T. turgidum* were promoted by seed coating with PGPB *Paraburkholderia phytofrmans* under water-nutrient stress [204].

#### 3.8.3 Biological control

Microbial inoculation to soils in the plant ecosystem can help decrease disease damage [205]. The biological potential of *Bacillus thuringiensis, Rhizobium meliloti, Aspergillus niger*, and *Trichoderma harzianum* has been evaluated through seed coating with gum arabic, glucose, sugar, and molasses in the suppression of root rot fungi (e.g., *Rhizoctonia solani* and *Fusarium* sp.) on *Helianthus annuus* and *Abelmoschus esculentus*. For instance, seed dressing of microbial antagonists e.g., *B. thuringiensis, R. meliloti* and *T. harzianum* improved the microbial efficiency in the control of root rot fungi on crop plants [150]. Also, it has been reported that the growth parameters such as shoot and root length, shoot and root weight considerably boosted in *A. esculentus* and *H. annuus* plants when seeds were treated with microorganisms, whereas no considerable varieties were perceived in the germination of seed treated by sugar, molasses, glucose, and gum Arabic [206]. The research was carried out to appraise the impact of seed coating with biological agents on the seed quality of rice. In this study, isolates of *Pseudomonas and Bacillus subtilis* were tested against *Xanthomonas oryzae* pv. *Oryzae.* Results showed that treatments of biological control boosted seed vigor, and reduced infection of *Xanthomonas oryzae* pv. *Oryzae* in the seed [207]. To reduce aflatoxin contamination in corn kernels, the biocontrol techniques were performed via film coating. The findings demonstrated that seeds coated with conventional pesticides such as insecticide (e.g., imidacloprid), fungicide (e.g., metalaxyl-M), and spores of non-aflatoxigenic *Aspergillus flavus* NRRL 30,797 reduced aflatoxin contamination of kernels [163]. Lately, it has been found that seed coating and soil drenching with three biocontrol bacterial strains (e.g., strains (e.g., *Providencia vermicola* and *Pseudomonas fluorescens*) boosted cucumber yield and decreased nematode infestation [208]. A biological investigation demonstrated that coating the seeds with the formulation of hydrogel, *Trichoderma harzianum*, and *Burkholderia gladioli* could protect *Phaseolus vulgaris* against common phytopathogens and improve seed germination [209]. Coating seeds of *Triticum durum* with sixty-two rhizosphere and endophytic bacterial strains caused the blockage of growth and germination *Fusarium culmorum* [172]. It has been proved that biological agents used in rice seed coating could improve the seed quality, seedling growth and decrease the blast disease to 0% [207]. Seed coating with entomopathogenic fungi *Metarhizium* sp., and *Beauveria* sp. protected seedlings of *Z. mays* against herbivorous insects by enhancing salicylic acid, and jasmonic acid contents [194].

#### 3.8.4 Ecological restoration by a beneficial microorganism

Restoration of ecology is a process that helps the recovery of degraded, damaged, or destroyed ecosystems. It is well known that PBM and their interactions with plants play an important part in the confirmation of ecological vegetation and sustaining physical structures in soils and nutrient cycling [210]. Seed coating with PBM can reduce challenges regarding soil moisture variables, low soil nutrients, pathogens in the environment [211]. For instance, the inoculation of *Aspergillus* sp. and *Streptomyces* sp. via seed coating improved the emergence of seedlings and survival of *Lolium multiflorum* and *Astragalus sinicus* on degraded rangeland in the Qinghai–Tibetan Plateau [212]. The use of combination *P. libanensis* and *R. irregularis* in seed coating of cowpea not only enhanced the production of crops but also improved soil fertility and seedlings tolerance against environmental stresses [154]. Indeed, the application of PBM can be a suitable tool for the sustainable production of crops and enhancement of yield and ecological restoration under different environmental conditions.

## 4 Future perspectives and challenges of seed coating

Seed coating is a method that can improve the germination index and seedling establishment in small and tiny seeds, and it reduces seed wastage. In this method, the physical properties of the seeds can be improved so that seed planting can be carried out easily, and the uniformity of the seedling emergence can be achieved, enhancing marketability in some plants, including vegetables. In addition, it can protect the seeds from being eaten by insects and animals that live in the soil. In the past, coating technology employed human health and environmentally hazardous chemicals. However, the recent developments in the use of biological agents are considered a new coating technology method, which, in addition to the beneficial environmental effects, can strengthen the seed performance and the physiological properties of the plants. In this technology, the used seeds must have optimum physical properties since seeds impurities, including broken seeds, other cultivars, and seeds of other plants, can cause problems in final germination. In the conditions of abiotic stresses, such as drought and salinity, it is possible to use moisture-absorbent compounds in seed coating so that the plant can be appropriately established in these conditions. Also, using microorganisms such as PGPR and PGPF can be beneficial in dealing with abiotic stressors, both specifically and in general, and improve the plant's antioxidant defense system against pathogens. Seeds primed with biological or non-biological materials undergo physiological steps before the emergence of the root and, compared to non-primed seeds, have a low storability. Therefore, farmers must have faster access to these (coated) seeds in order to have beneficial effects. In other words, the interval between covering the seeds and planting them in the field should be done quickly. Additionally, in the coating technology, the optimal population of microorganisms should also be considered because their low population does not have the necessary efficiency to improve growth and their high population also creates a competitive environment. For example, seed inoculation requires 10^8^ colonies per ml of distilled water for population of *Pseudomonas fluorescens* [16,213] and 10^7^ spores per ml of distilled water for the fungal population of *Trichoderma harzianum* [214]. The use of superabsorbent materials such as vermiculite, kaolin, and perlite can be suitable carriers for microorganisms because these compounds not only have sufficient porosity and space to absorb moisture and the growth and survival of microorganisms around the seed but also these substances do not prevent the radicle emergence of seeds, which leads to an optimal establishment of the seedling.

## 5 Conclusions and future scenarios

Seed coating is a technique of covering seeds to improve plant establishment and growth, and protect plants against biotic (e.g., pests and diseases) and unfavorable environmental conditions (e.g., drought, salinity, and extreme temperatures), thus providing a secure environment for the next generations. Indeed, the seed coating process is a suitable technology in sustainable agriculture that has received attention today.

Several experimental underlines about microbial seed coating as a biotechnological reach to meliorate crop yield and quality against environmental stress. However, large-scale application and broader use of seed coating have been hindered by several parameters such as survival and viability of microorganisms, selection of the ingredient and accurate formulation, and production cost, which need to be identified by more studies. Also, it is considered that the advantages of microbial seed coating for its application in agriculture are not always assured since it varies with plant species, conditional growth, and experimental scale. However, nowadays using seed coating and efficient PBM strains in agricultural production can provide a commercial market.

The future of seed coating is dependent on formulations, which should be adjusted according to the local conditions and agriculture practices (such as the application of pesticides, fertilizers, and irrigation management). Known PBM formulations obtained by native strains under local conditions need to be further explored. The efficient formulations improve not only the survival of PBM but also the growth and performance of plants. Considering climate changes, the performance of PBM demonstrates in reduction of biotic and abiotic stress. Therefore, the application of PBM in seed coating is promising, and it has great potential for agricultural practice in the future. PBM seed coating is an efficient tool for sustainable agriculture that needs more expansion and investiture to provide its widespread implementation and integration in agricultural management strategies.

## CRediT authorship contribution statement

**Arezoo Paravar:** Resources, Writing – original draft. **Ramin Piri:** Resources, Writing – original draft. **Hamidreza Balouchi:** Supervision, Writing – review & editing. **Ying Ma:** Supervision, Writing – review & editing.

## Declaration of Competing Interest

The authors declare that they have no known competing financial interests or personal relationships that could have appeared to influence the work reported in this paper.

## Data Availability

No data was used for the research described in the article.
